# Nonselective TRPC channel inhibition and suppression of aminoglycoside-induced premature termination codon readthrough by the small molecule AC1903

**DOI:** 10.1016/j.jbc.2021.101546

**Published:** 2022-01-06

**Authors:** Alireza Baradaran-Heravi, Claudia C. Bauer, Isabelle B. Pickles, Sara Hosseini-Farahabadi, Aruna D. Balgi, Kunho Choi, Deborah M. Linley, David J. Beech, Michel Roberge, Robin S. Bon

**Affiliations:** 1 Department of Biochemistry and Molecular Biology, Life Sciences Institute, The University of British Columbia, Vancouver, British Columbia, Canada; 2 Discovery and Translational Science Department, Leeds Institute of Cardiovascular and Metabolic Medicine, School of Medicine, University of Leeds, Leeds, UK; 3 School of Chemistry, University of Leeds, Leeds, UK; 4 Astbury Centre for Structural Molecular Biology, University of Leeds, Leeds, UK

**Keywords:** genetic disease, premature termination codon readthrough, aminoglycoside, G418, ion channel, TRP channel, AC1903, Pico145, translation, 2-APB, 2-aminoethoxydiphenylborane, 4α-PDD, 4α-phorbol 12,13-didecanoate, [Ca^2+^]_i_, intracellular concentration of Ca^2+^, EA, (-)-englerin A, HEK T-REx cells, human embryonic kidney 293 cells stably expressing the tetracycline repressor protein, OAG, 1-oleoyl-2-acetyl-sn-glycerol, PTC, premature termination codon, S1P, sphingosine-1-phosphate, TRPC, transient receptor potential canonical

## Abstract

Nonsense mutations, which occur in ∼11% of patients with genetic disorders, introduce premature termination codons (PTCs) that lead to truncated proteins and promote nonsense-mediated mRNA decay. Aminoglycosides such as G418 permit PTC readthrough and so may be used to address this problem. However, their effects are variable between patients, making clinical use of aminoglycosides challenging. In this study, we tested whether TRPC nonselective cation channels contribute to the variable PTC readthrough effect of aminoglycosides by controlling their cellular uptake. Indeed, a recently reported selective TRPC5 inhibitor, AC1903, consistently suppressed G418 uptake and G418-induced PTC readthrough in the DMS-114 cancer cell line and junctional epidermolysis bullosa (JEB) patient-derived keratinocytes. Interestingly, the effect of AC1903 in DMS-114 cells was mimicked by nonselective TRPC inhibitors, but not by well-characterized inhibitors of TRPC1/4/5 (Pico145, GFB-8438) or TRPC3/6/7 (SAR7334), suggesting that AC1903 may work through additional or undefined targets. Indeed, in our experiments, AC1903 inhibited multiple TRPC channels including TRPC3, TRPC4, TRPC5, TRPC6, TRPC4–C1, and TRPC5–C1, as well as endogenous TRPC1:C4 channels in A498 renal cancer cells, all with low micromolar IC_50_ values (1.8–18 μM). We also show that AC1903 inhibited TRPV4 channels, but had weak or no effects on TRPV1 and no effect on the nonselective cation channel PIEZO1. Our study reveals that AC1903 has previously unrecognized targets, which need to be considered when interpreting results from experiments with this compound. In addition, our data strengthen the hypothesis that nonselective calcium channels are involved in aminoglycoside uptake.

Nonsense mutations account for ∼11% of all genetic lesions in patients with inherited diseases (1). They introduce a TAG, TGA, or TAA premature termination codon (PTC) and typically result in production of mRNAs with decreased stability as well as defective truncated proteins. PTC readthrough is a mechanism by which ribosomes recognize nonsense mutations as sense codons, enabling synthesis of the full-length and functional protein rather than truncated product. Aminoglycoside antibiotics were the first and are still among the most active chemicals discovered to induce PTC readthrough in yeast (2), mammalian cells (3), animal models (4, 5), and patients (6, 7). These compounds bind at the decoding center of eukaryotic ribosomes and facilitate pairing of near-cognate aminoacyl-tRNAs to the PTCs resulting in formation of full-length protein (8, 9). Several nonaminoglycoside readthrough compounds have also been identified, including negamycin, tylosin, RTC13, RTC14, GJ71, GJ72, and ataluren (10, 11, 12, 13, 14). However, these compounds induce PTC readthrough at low rates and often at or below the detection limit of Western blotting for endogenous protein expression.

In addition to their severe *in vivo* toxicity, aminoglycosides induce variable levels of PTC readthrough in cell lines as well as patients, making long-term clinical administration of these drugs challenging (15, 16, 17, 18, 19, 20). In general, variation in drug response is caused by differential local drug concentrations (pharmacokinetics) or drug actions (pharmacodynamics) and to a great extent is attributed to genetic variations (21). Variations in aminoglycoside-induced PTC readthrough could be in part related to the PTC and its surrounding sequence as well as its distance to the poly-A tail sequence. Moreover, differential cellular uptake of aminoglycosides and consequent variable intracellular concentrations of these compounds may underlie mechanisms of PTC readthrough variation. However, the correlation of genetic variations in genes involved in cellular uptake of aminoglycosides and their contribution to PTC readthrough in human cells is not well understood.

Endocytosis (22, 23) and permeation through nonselective cation channels (including TRP channels) (24, 25, 26, 27, 28) are the main proposed mechanisms for cellular uptake of aminoglycosides. In this study, we addressed the hypothesis that TRPC channels may contribute to variable effects of aminoglycoside-mediated PTC readthrough. Following the observation that low PTC readthrough in response to treatment with the aminoglycoside G418 (which is cationic at physiological pH) was correlated with a mutation in *TRPC5*, we tested the effects of selected TRPC channel inhibitors on G418-induced PTC readthrough. We found that the 2-aminobenzimidazole derivative AC1903, recently reported as a selective TRPC5 channel inhibitor (29, 30), suppresses cellular uptake of the aminoglycoside G418 and G418-induced PTC readthrough in the DMS-114 cell line and in JEB patient-derived keratinocytes. PTC readthrough suppression in DMS-114 cells could be reproduced with the nonselective calcium channel modulators SKF96365 and 2-APB, but not with the well-characterized, selective TRPC1/4/5 inhibitors Pico145 and GFB-8438, suggesting that AC1903 may have additional targets. We subsequently found that, in contrast to previous reports, AC1903 is not a selective inhibitor of TRPC5 (or TRPC1/4/5) channels, but that it inhibits multiple TRP channels, including those formed by TRPC3, TRPC4, TRPC6, and TRPV4. Our study underlines that investigations of TRPC5 channel biology with nonselective inhibitors such as AC1903 need to be supported carefully by orthogonal chemical or genetic approaches. In addition, our results are consistent with the hypothesis that multiple nonselective (TRP) cation channels can mediate cellular aminoglycoside uptake, which may trigger new investigations into the use of aminoglycosides for the treatment of genetic diseases.

## Results

### Variable aminoglycoside-induced PTC readthrough in cell lines with identical *TP53* nonsense mutation

**Figure 1:**
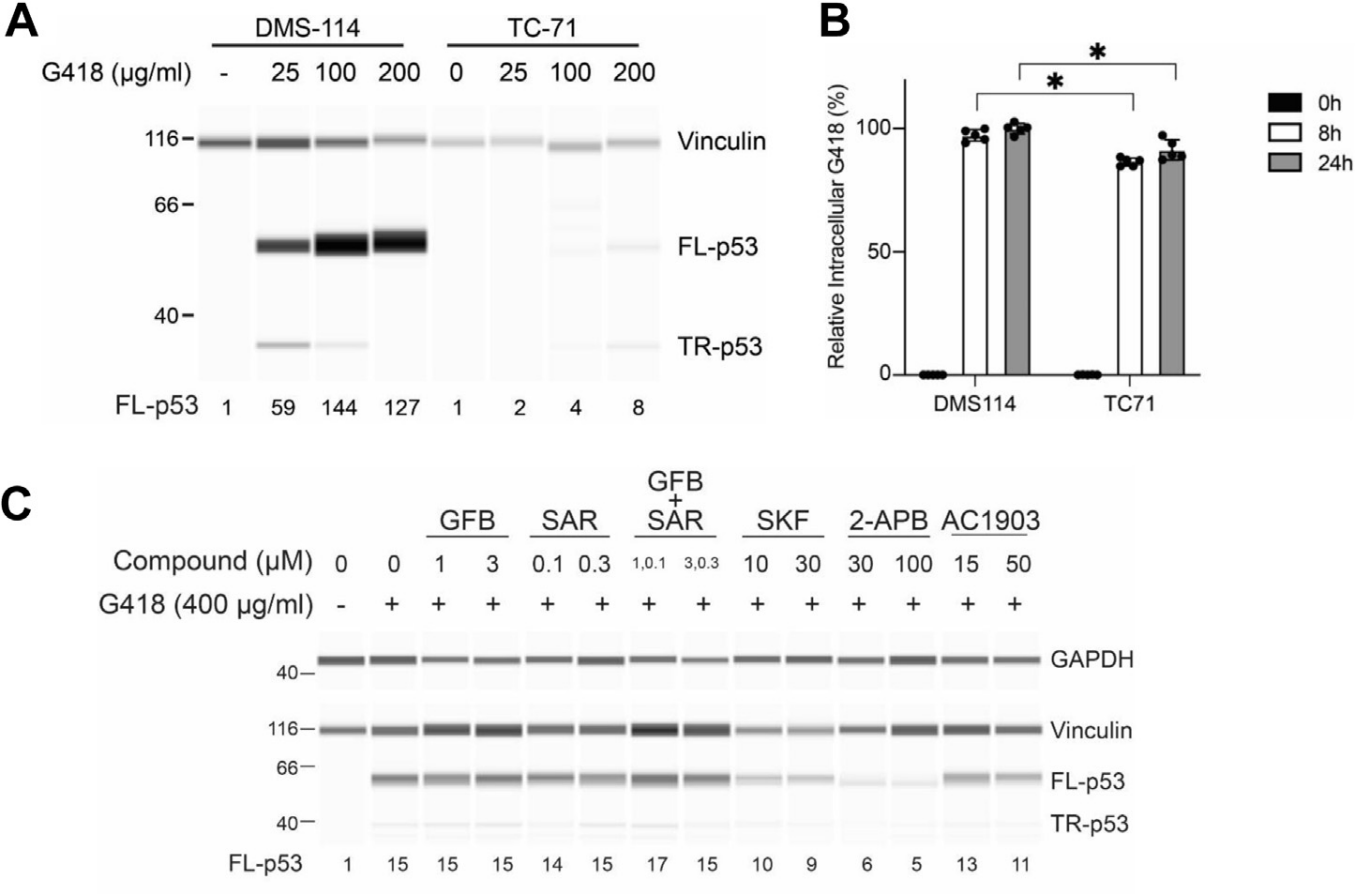
**Effect of TRPC inhibitors on G418 induced PTC readthrough**. *A*, DMS-114 and TC-71 cell lines with homozygous *TP53* nonsense mutation (R213X) were exposed to indicated concentrations of G418 for 24 h and p53 levels (full-length, FL-p53; truncated, TR-p53) were measured. *B*, 100 μg/ml G418 was added to DMS-114 and TC-71 cells and its total intracellular levels were measured at indicated time points (n = 5; data normalized to the highest response in each experiment; bars represent mean ± SD). *C*, DMS-114 cells were preincubated with indicated concentrations of TRPC inhibitors GFB-8438 (GFB), SAR7334 (SAR), SKF96365 (SKF), 2-APB, or AC1903 for 3 h followed by exposure to 400 μg/ml G418 for another 3 h. At 24 h cell lysates were prepared and p53 levels were measured. In panels *A* and *C*, protein levels were measured by automated capillary electrophoresis Western analysis (using vinculin and GAPDH as loading controls). Samples of cell lysates containing equal amounts of total protein were loaded in all capillaries and FL-p53 levels were expressed relative to the amount of FL-p53 in untreated cells. ∗ indicates statistically significant difference between samples according to two-way ANOVA (*p* < 0.05). PTC, premature termination codon; TRPC, transient receptor potential canonical.

Variation in PTC readthrough response has been reported among individuals or cell lines with different nonsense mutations. Although this in part highlights the importance of the stop codon and its surrounding sequence as well as the physical location of the nonsense mutation in the mRNA sequence, understanding the contribution of other underlying genetic variations becomes more complicated. To rule out the effect of the sequence and position of nonsense mutations on response variation to potential PTC readthrough modulators, we studied two cancer cell lines, DMS-114 and TC-71, with identical homozygous nonsense mutation R213X in their *TP53* gene. Exposure of DMS-114 cells to increasing concentrations of the aminoglycoside G418 for 24 h resulted in strong, concentration-dependent increase in production of full-length p53 (the readthrough product) up to 144-fold compared with untreated cells (Fig. 1*A*). In contrast, increasing concentrations of G418 only elicited a small increase in PTC readthrough in TC-71 cells, up to eightfold compared to untreated cells (Fig. 1*A*). To find out whether the reduced response to G418 in TC-71 cells was associated with lower intracellular levels of G418, we measured concentrations of G418 in both cell lines at 0, 8 and 24 h post exposure to G418 (100 μg/ml). Compared with DMS-114, intracellular G418 levels were slightly but significantly lower in TC-71 cells at 8 h and 24 h post G418 exposure, by ∼11% and ∼9%, respectively (Fig. 1*B*). These data suggest that reduced intracellular G418 level in TC-71 cells *versus* DMS-114 may be partially responsible for the difference in G418-induced PTC readthrough.

To find out the possible contribution of genetic variants to this variation, we searched exome sequencing data in COSMIC (https://cancer.sanger.ac.uk/cosmic) and identified a hemizygous p.R175H missense mutation in *TRPC5* in the TC-71 cell line not present in the DMS-114 cell line. This gene encodes the protein TRPC5, which forms homo- and heterotetrameric, nonselective cation channels permeable by Ca^2+^ and Na^+^ (31, 32, 33, 34, 35, 36, 37). Based on several pathogenicity prediction algorithms including SIFT (38) and PolyPhen-2 (39), this mutation is considered tolerable and benign. R175 of TRPC5 is located in a putative zinc-binding domain—consisting of H172, C176, C178, and C181—that is conserved between TRPC proteins (40, 41). This domain has also been implicated in TRPC5 regulation by S-glutathionylation (42), as well as S-palmitoylation required for TRPC5 trafficking to the plasma membrane (43). Because other nonselective cation channels of the TRP family have been reported to contribute to aminoglycoside import (24, 25, 26, 27, 28), we decided to test the potential role of TRPC5 and other TRPC channels in this process.

### Identification of AC1903 as a suppressor of G418-mediated PTC readthrough

To test whether TRPC5 or other TRPC channels may contribute to G418-mediated PTC readthrough, we screened a panel of TRPC inhibitors in DMS-114 cells. To this end, we selected compounds reported as selective TRPC channel inhibitors: AC1903 (TRPC5) (29, 30); GFB-8438 (TRPC4/5) (44); or SAR7334 (TRPC3/6/7) (45). For the simultaneous inhibition of all TRPC channels, we either used a combination of GFB-8438 and SAR7334, or the nonselective calcium channel modulators SKF96365 or 2-APB, each of which affects a broad spectrum of TRP channels and inhibits TRPC channels. Compounds were screened at two concentrations, reflecting their relative potencies against various TRPC channels. In our experiments, PTC readthrough induced by 400 μg/ml G418 was suppressed by SKF96365 (∼33%) and especially by 2-APB (∼66%), but GFB-8438, SAR7334, or their combination had no effect (Fig. 1*C*). Interestingly, AC1903 had an effect similar to SKF96365 when used at 50 μM (*i.e.*, about three times its reported IC_50_ for TRPC5 currents (29)), suppressing G418-induced PTC readthrough by ∼27% (Fig. 1*C*). Therefore, we decided to investigate the effects of AC1903 in more detail.

### AC1903 suppresses G418 trafficking and PTC readthrough in DMS-114 cell line and JEB patient-derived keratinocytes

**Figure 2:**
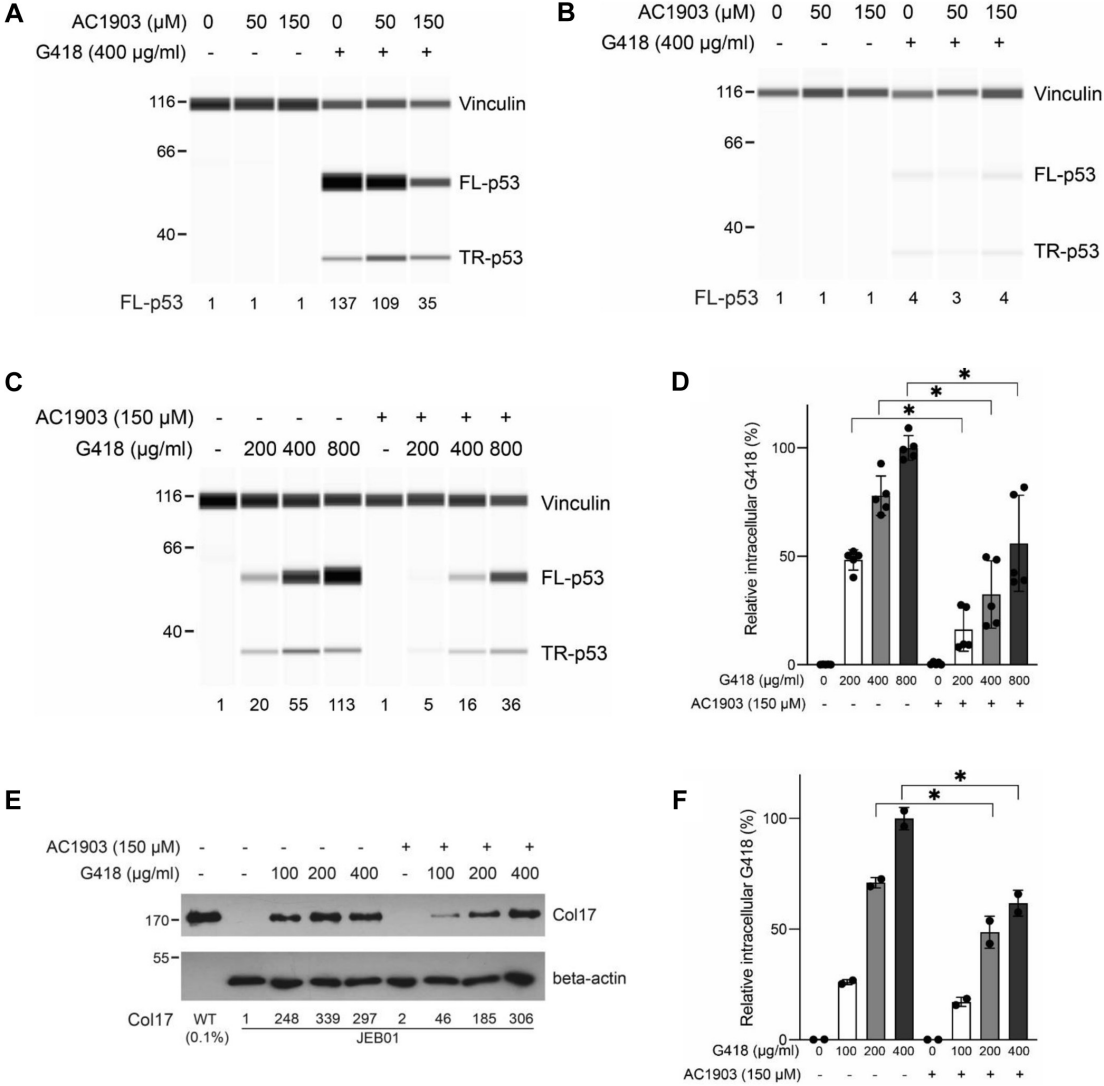
**Effect of AC1903 on PTC readthrough and G418 trafficking.***A* and *B*, DMS-114 (*A*) or TC-71 (*B*) cells were pretreated with indicated concentrations of AC1903 for 3 h followed by exposure to 400 μg/ml G418. After 3 h medium was replenished and at 24 h cell lysates were prepared and p53 levels were measured. *C* and *E*, DMS-114 (*C*) or JEB01 (*E*) cells were either left untreated or pretreated with 150 μM AC1903 for 3 h followed by exposure to indicated concentrations of G418 for another 3 h. At 24 h cell lysates were prepared and p53 (*C*) or Collagen XVII (*E*) levels were measured. In panels *A*–*C*, protein levels were measured by automated capillary electrophoresis Western analysis (using vinculin as loading control). In panel *E*, protein levels were measured by Western blot (using beta-actin as loading control). Samples of cell lysates containing equal amounts of total protein were loaded in all capillaries and FL-p53 or Collagen XVII levels were expressed relative to their respective amounts in untreated cells. *D*, total intracellular levels of G418 were measured in the same lysates as in (*C*) (n = 5; data normalized to the highest response in each experiment; bars represent mean ± SD). *F*, total intracellular levels of G418 were measured in the same lysates as in (*E*) (n = 2; data normalized to the highest response in each experiment; bars represent mean ± SD). ∗ indicates statistically significant difference between samples according to one-way ANOVA (*p* < 0.05). PTC, premature termination codon.

To further investigate AC1903 as a suppressor of G418-induced PTC readthrough, we exposed DMS-114 and TC-71 cells to various concentrations of AC1903 for 3 h prior to exposure to 400 μg/ml G418 for another 3 h, followed by media replacement and incubation for another 18 h. Exposure to AC1903 alone did not affect PTC readthrough in either cell line (Fig. 2, *A* and *B*). As expected, G418 elicited strong PTC readthrough in DMS-114 but not in TC-71 cells (Fig. 2, *A* and *B*). In DMS-114 but not in TC-71 cells, combination of AC1903 (50 or 150 μM) with G418 reduced PTC readthrough by 20% and 74%, respectively, compared with G418 alone (Fig. 2, *A* and *B*).

In order to better understand the effects of AC1903 on G418 uptake and PTC readthrough, we exposed DMS-114 cells and JEB01 keratinocytes, derived from a junctional epidermolysis bullosa (JEB) patient with a homozygous nonsense mutation (p.R688X) in the *COL17A1* gene, to increasing concentrations of G418 without or with pretreatment with 150 μM AC1903. Exposure of DMS-114 cells to G418 alone resulted in a concentration-dependent increase in full-length p53, which decreased ∼70% when cells were additionally treated with AC1903 (Fig. 2*C*). This reduction in PTC readthrough was correlated with significantly decreased intracellular G418 levels in the presence of AC1903 (Fig. 2*D*). Similarly, JEB01 keratinocytes produced full-length Collagen XVII in the presence of G418 alone, which declined ∼45–80% in combination with AC1903 (Fig. 2*E*). Again, reduction of PTC readthrough in JEB01 cells exposed to AC1903 was correlated with significantly declined intracellular G418 levels (Fig. 2*F*).

These data suggest that AC1903 suppresses cellular uptake of G418 and G418-induced PTC readthrough in DMS-114 and JEB01 cells, and the findings could indicate a possible role of TRPC5 channels in cellular regulation of G418 uptake. However, because the effect of AC1903 on G418-induced PTC readthrough could not be reproduced by the TRPC4/5 channel inhibitor GFB-8438 (Fig. 1*C*), further investigation was required.

### The TRPC1/4/5 inhibitor Pico145 does not modulate G418 uptake and G418-induced PTC readthrough in DMS-114 cells

**Figure 3:**
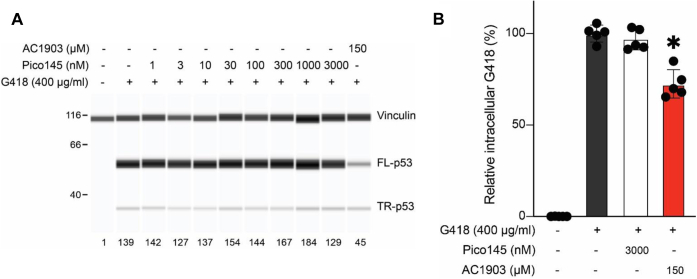
**Effect of the selective TRPC1/4/5 inhibitor Pico145 on G418-induced PTC readthrough.***A* and *B*, DMS-114 cells were preincubated with 150 μM AC1903 or indicated concentrations of Pico145 for 3 h followed by exposure to 400 μg/ml G418 for another 3 h. At 24 h cell lysates were prepared and p53 were measured by automated capillary electrophoresis Western analysis (using vinculin as loading control) (*A*). Samples of cell lysates containing equal amounts of total protein were loaded in all capillaries and FL-p53 levels were expressed relative to the amount of FL-p53 in untreated cells. In selected cell lysates, intracellular G418 levels (*B*) were measured (n = 5; data normalized to the vehicle control in each experiment; bars represent mean ± SD). ∗ indicates statistically significant difference between samples according to one-way ANOVA (*p* < 0.05). TRPC, transient receptor potential canonical.

In an attempt to further probe the potential role of TRPC5 channels in the regulation of intracellular G418 levels by AC1903, we exposed DMS-114 cells to the xanthine derivative Pico145 in the presence of 400 μg/ml G418. Pico145 (also named HC-608) is a well-characterized, highly selective chemical probe of TRPC5, TRPC4, and heteromeric TRPC1/4/5 channels with picomolar to nanomolar potency (40, 46, 47, 48) that has been used successfully to inhibit endogenous TRPC1/4/5 channels in cells (46, 49, 50, 51, 52), tissues (50, 53, 54), and animals (47, 54, 55). Unlike AC1903, Pico145 (up to 3 μM) did not affect PTC readthrough or intracellular G418 levels (Fig. 3, *A* and *B*). These findings suggest that TRPC1/4/5 channel inhibition is not sufficient to mimic AC1903 as a suppressor of G418-induced PTC readthrough in DMS-114 cells.

### AC1903 is not a selective TRPC5 channel inhibitor

Based on the findings described above, we wondered whether AC1903—which is typically used as a TRPC5 inhibitor at concentrations of 30–100 μM in cellular assays (29, 56)—might have limited selectivity for TRPC5 channels and whether its effect on intracellular G418 levels and PTC readthrough might be the consequence of inhibition of other or multiple targets. All well-characterized TRPC channel inhibitors reported to date modulate multiple TRP(C) channels (34, 35, 36). Indeed, screening data deposited in PubChem by the John Hopkins Ion Channel Center in 2010 suggest that AC1903 can also inhibit TRPC4 channels (IC_50_ 0.6 μM; PubChem Identifiers AID 434942 – SID 865888). Because limited data for the selectivity of AC1903 are available in the public domain, we decided to profile AC1903 against a selection of calcium-permeable cation channels by intracellular calcium ([Ca^2+^]_i_) recordings using well-characterized cell lines and TRP channel activators (46, 57, 58), and the Ca^2+^ indicator Fura-2. In these assays, cells (HEK 293, HEK T-REx, or CHO) overexpressing the relevant ion channel protein and loaded with Fura-2 AM (a cell-permeable precursor of Fura-2) were preincubated with various concentrations of AC1903 or vehicle (DMSO) for 30 min. Then, changes in ratiometric fluorescence (from background) were recorded upon addition of the respective ion channel activator.

**Figure 4:**
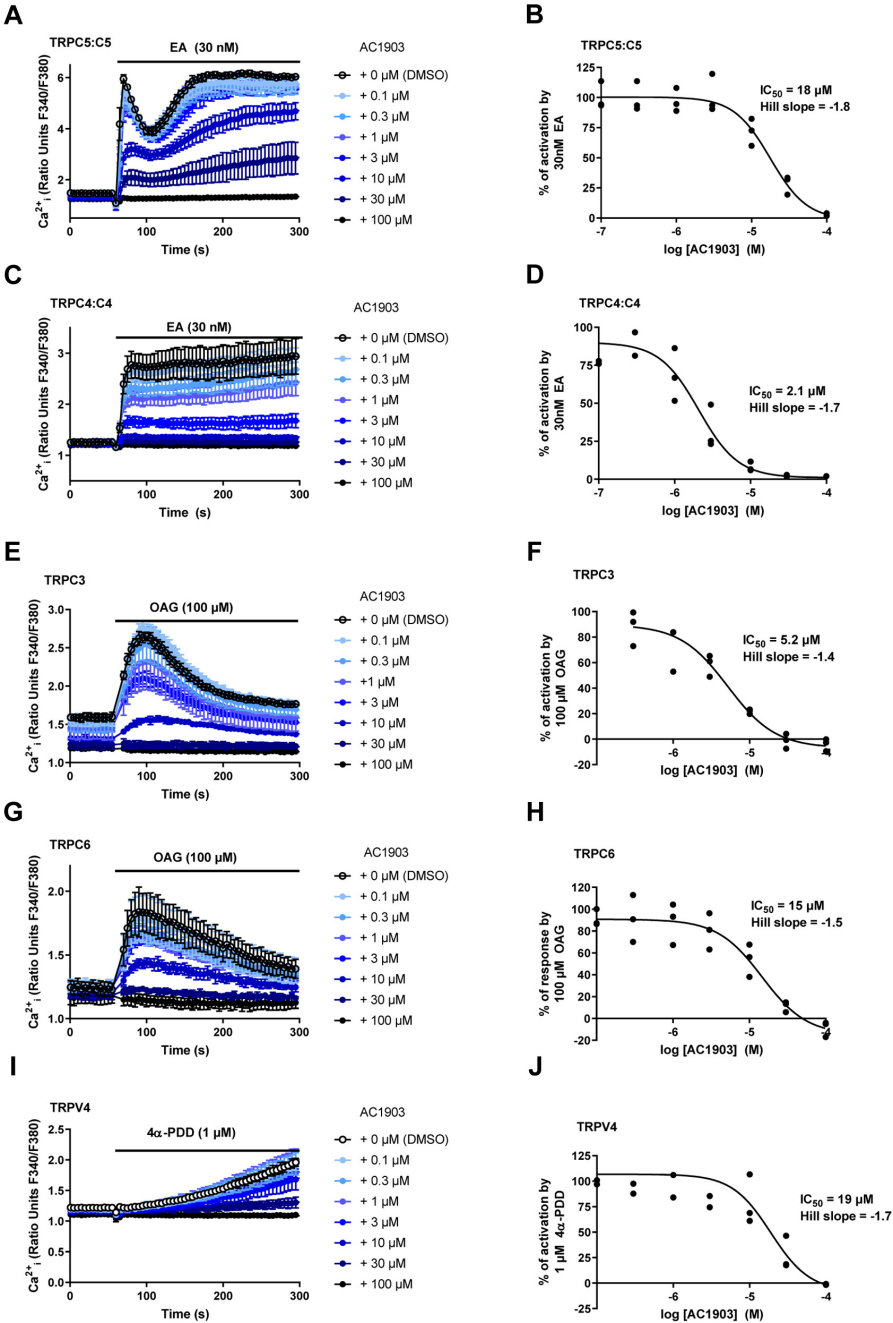
**AC1903 inhibits multiple TRP channels.***A* and *C*, representative [Ca^2+^]_i_ measurements from a single 96-well plate (N = 6; mean ± SD over technical replicates) showing inhibition of EA-mediated [Ca^2+^]_i_ responses by 0.1 to 100 μM AC1903 in (Tet+) HEK T-REx cells expressing TRPC5 (*A*) or TRPC4 (*C*). *B* and *D*, concentration–response data for experiments in (*A*) and (*C*) (scatter plots showing normalized data for three independent experiments; n/N = 3/18). Responses were calculated at 250 to 295 s compared to [Ca^2+^]_i_ at baseline (0–55 s). *E* and *G*, representative [Ca^2+^]_i_ measurements from a single 96-well plate (N = 6; mean ± SD over technical replicates) showing inhibition of OAG-mediated [Ca^2+^]_i_ responses by 0.1 to 100 μM AC1903 in (Tet+) HEK T-REx cells expressing TRPC3 (*E*) and WT HEK 293 cells transiently expressing TRPC6 (*G*). *F* and *H*, concentration–response data for experiments in (*E*) and (*G*) (scatter plots showing normalized data for three independent experiments; n/N = 3/18). Responses were calculated at 90 to 110 s compared with [Ca^2+^]_i_ at baseline (0–55 s). *I*, representative [Ca^2+^]_i_ measurements from a single 96-well plate (N = 6; mean ± SD over technical replicates) showing inhibition of the 4α-PDD-mediated [Ca^2+^]_i_ response by 0.1 to 100 μM AC1903 in CHO cells stably expressing TRPV4. *J*, concentration–response data for experiments in (*I*) (scatter plot showing normalized data for three independent experiments; n/N = 3/18). Responses were calculated at 290 to 300 s compared with [Ca^2+^]_i_ baseline (0–55 s). EA, (-)-englerin A; TRPC, transient receptor potential canonical.

**Figure 5:**
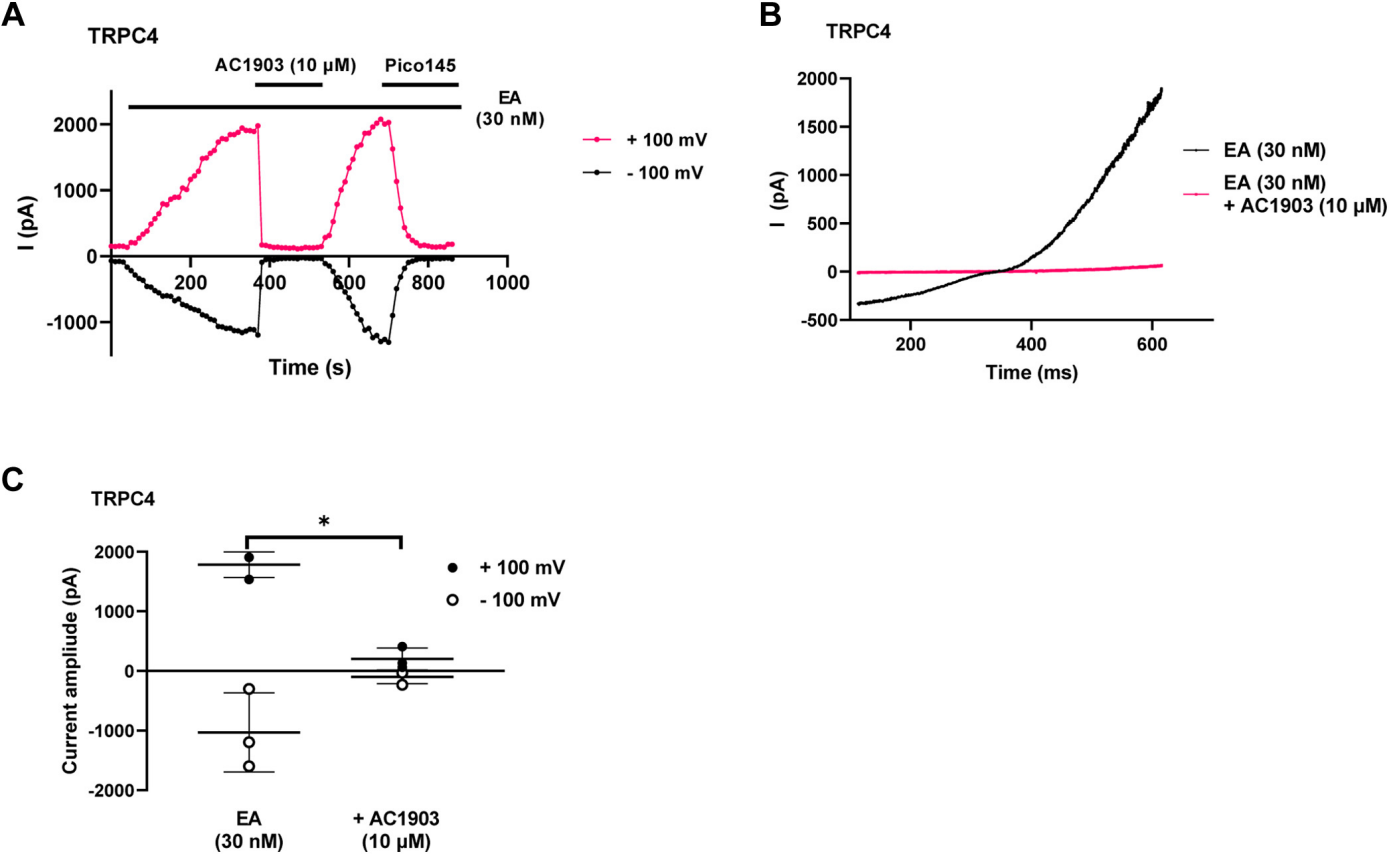
**AC1903 reversibly inhibits EA-activated TRPC4 currents in whole-cell patch-clamp recordings.***A*, representative trace from one (Tet+) HEK T-Rex cell expressing TRPC4, showing current at +100 mV (*magenta*) and −100 mV (*black*) after activation by EA (30 nM), followed by addition and washout of AC1903 (10 μM) and subsequent addition of Pico145. B) Representative current–voltage relationship for one (Tet+) HEK T-Rex cell expressing TRPC4, showing current after activation by EA (30 nM) and subsequent inhibition by addition of AC1903 (10 μM). *C*, quantified data from experiments represented by (*A*) (scatter plots showing current amplitudes at +100 mV (*black circles*) or −100 mV (*white circles*) from three independent experiments; bars represent mean ± SD; n = 3). ∗ indicates statistically significant difference between samples according to paired *t* test (*p* < 0.05). EA, (-)-englerin A; TRPC, transient receptor potential canonical.

We first established that our sample of AC1903 was analytically pure (Figs. S1–S4) and that AC1903 does not interfere with the optical properties of Fura-2 (Fig. S5). Then, we confirmed that AC1903 inhibits TRPC5:C5 channels activated by 30 nM (-)-englerin A (EA, a highly selective and efficacious TRPC1/4/5 activator (57)) (IC_50_ 18 μM) (Fig. 4, *A* and *B*) or 10 μM sphingosine-1-phosphate (S1P), a physiological TRPC4/5 agonist (IC_50_ 3.5 μM) (Fig. S6, *A* and *B*). To test the effect of AC1903 on TRPC4 channels, we initially attempted to use carbachol to activate TRPC4:C4 channels, in line with patch-clamp experiments reported by Zhou *et al.* (29). However, application of carbachol also resulted in an increase in [Ca^2+^]_i_ in wild-type HEK 293 cells that lacked a response to EA, and AC1903 inhibited this carbachol-mediated calcium response (IC_50_ 20 μM) (Fig. S7). Therefore, we used the selective TRPC1/4/5 activator EA and the physiological TRPC4/5 activator S1P instead. Consistent with data available on PubChem (see above), AC1903 inhibited TRPC4:C4 channels activated by 30 nM EA (IC_50_ 2.1 μM) (Fig. 4, *C* and *D*) or 10 μM S1P (IC_50_ 1.8 μM) (Fig. S6, *C* and *D*). These results were confirmed by whole-cell patch-clamp recordings in TRPC4-expressing HEK T-REx cells, in which EA-activated currents were fully and reversibly inhibited by 10 μM AC1903 (Fig. 5). An additional experiment with cumulative addition of AC1903 (0.3–30 μM) showed concentration-dependent inhibition of EA-activated TRPC4 currents (Fig. S8). AC1903 also inhibited EA-induced Ca^2+^ influx mediated by channels formed by concatemeric TRPC5–C1 (IC_50_ 4.7 μM) (Fig. S9, *A* and *B*) and TRPC4–C1 (IC_50_ 3.0 μM) (Fig. S9, *C* and *D*) (46, 58, 59, 60), suggesting that AC1903 nonselectively inhibits TRPC1/4/5 channels. AC1903 also inhibited EA-mediated Ca^2+^ influx in A498 cells, which express endogenous TRPC1:C4 channels (IC_50_ 3.4 μM) (Fig. S9, *E* and *F*). AC1903 on its own did not affect viability of A498 cells (Fig. S10*A*), but—consistent with inhibition of TRPC1:C4 channels (59)—AC1903 (at 100 μM) inhibited EA-mediated A498 cytotoxicity (Fig. S10*B*).

Further profiling studies showed that AC1903 can also inhibit OAG-activated channels formed by TRPC3 (IC_50_ 5.2 μM) (Fig. 4, *E* and *F*) and TRPC6 (IC_50_ 15 μM) (Fig. 4, *G* and *H*), as well as 4α-PDD-activated TRPV4 channels (IC_50_ 19 μM) (Fig. 4, *I* and *J*). In contrast, concentration of up to 30 μM of AC1903 did not inhibit capsaicin-activated TRPV1 or Yoda1-activated Piezo1 channels, but small effects were seen at 100 μM AC1903 (Fig. S11). The combined data (summarized in Table 1) suggest that AC1903 can inhibit a range of TRP channels with similar (low micromolar) potencies, but that TRPV1 is relatively resistant.

**Table 1:** Summary of selectivity profiling of AC1903

Ion channel (cell type)	Activator (concentration)	IC_50_ (comment)	Data figures
TRPC5:C5 (HEK T-REx)	EA (30 nM)	18 μM	Figure 4, *A* and *B*
TRPC5:C5 (HEK T-REx)	S1P (10 μM)	3.5 μM	Fig. S6, *A* and *B*
TRPC5–C1 (HEK T-REx)	EA (30 nM)	4.7 μM	Fig. S9, *A* and *B*
TRPC4:C4 (HEK T-REx)	EA (30 nM)	2.1 μM (consistent with patch-clamp data)	Figure 4, *C* and *D* Figure 5 Fig. S8
TRPC4:C4 (HEK T-REx)	S1P (10 μM)	1.8 μM	Fig. S6, *C* and *D*
TRPC4–C1 (HEK T-REx)	EA (30 nM)	3.0 μM	Fig. S9, *C* and *D*
TRPC1:C4 (A498)	EA (100 nM)	3.4 μM (consistent with inhibition of EA-induced cytotoxicity)	Fig. S9, *E* and *F* Fig. S10
TRPC3:C3 (HEK T-REx)	OAG (100 μM)	5.2 μM	Figure 4, *E* and *F*
TRPC6:C6 (HEK 293)	OAG (100 μM)	15 μM	Figure 4, *G* and *H*
TRPV1:V1 (HEK 293)	capsaicin (500 nM)	>50 μM	Fig. S11, *A* and *B*
TRPV4:V4 (CHO)	4α-PDD	19 μM	Figure 4, *I* and *J*
Piezo1 (HEK T-REx)	Yoda1 (3 μM)	>100 μM	Fig. S11, *C* and *D*
WT (HEK 293)	carbachol (100 μM)	20 μM	Fig. S7

### TRPV4 inhibition is not sufficient to suppress G418-induced PTC readthrough in DMS-114 cells

Nonselective cation channels formed by TRPV4 have been proposed to enable aminoglycoside uptake in kidney distal tubule cells (24) and hair cells in the inner ear (28). Since TRPV4 is among the ion channels inhibited by AC1903, we asked whether selective inhibition of this channel can suppress G418-induced PTC readthrough in DMS-114 cells. To address this, we exposed cells to either AC1903 (150 μM) or increasing concentrations (0.03–3 μM) of GSK2193874, a selective TRPV4 inhibitor (IC_50_ 40 nM for hTRPV4) (61, 62), for 3 h followed by incubation with 400 μg/ml G418. While 150 μM AC1903 strongly reduced G418-induced PTC readthrough, GSK2193874 alone or in combination with G418 did not have a major effect on full-length p53 production (Fig. S12), suggesting that TRPV4 channel inhibition is not sufficient to mimic the effect of AC1903 on G418-mediated PTC readthrough in DMS-114 cells.

## Discussion

In this study, we identified AC9103 as a suppressor of G418-induced PTC readthrough in DMS-114 cells and JEB patient-derived cells. We initially observed a remarkable difference in G418-induced readthrough of the *TP53* gene between DMS-114 and TC-71 cells, despite both cell lines having the same nonsense mutation in *TP53*. We also found slightly lower uptake of G418 in TC-71 cells compared with DMS-114 cells. Because we identified a missense mutation in *TRPC5* in the TC-71 cell line, and because nonselective cation channels (including TRP channels) have been implicated in cellular uptake of aminoglycosides, we screened a panel of TRPC channel inhibitors with different reported selectivities. We found that 2-APB and SKF96365—two broad-spectrum calcium channel modulators that inhibit TRPC channels—suppressed G418-induced PTC readthrough in DMS-114 cells. This effect was mimicked by the 2-aminobenzimidazole derivative AC1903—previously reported to be a selective TRPC5 channel inhibitor (29, 30). AC1903 (50–150 μM) suppressed both G418 uptake and subsequent PTC readthrough in the DMS-114 cell line, as well as in JEB01 keratinocytes derived from a JEB patient. However, the effects could not be reproduced with the selective, nanomolar TRPC1/4/5 inhibitors Pico145 and GFB-8438 (each used at concentrations up to 3 μM). These experiments suggested that TRPC1/4/5 inhibition is not sufficient to suppress G418 import in DMS-114 cells, and that AC1903 may have previously unrecognized targets. Indeed, a PubChem search suggested that AC1903 can also inhibit TRPC4 channels (PubChem Identifiers AID 434942 – SID 865888). This prompted us to profile AC1903 against a range of nonselective cation channels.

Our experiments confirmed that AC1903 can inhibit calcium entry mediated by TRPC5:C5 channels (activated with EA or S1P), as well as EA-activated concatemeric TRPC5–C1 channels, which have functional and pharmacological properties similar to endogenous TRPC1:C5 channels. However, in contrast to the claim that AC1903 is a selective TRPC5 inhibitor with respect to TRPC4 and TRPC6 channels (29, 30), our data suggest that AC1903 can also inhibit calcium entry mediated by TRPC4:C4, TRPC4–C1, TRPC3:C3, and TRPC6:C6 channels, with IC_50_ values similar to or lower than those for TRPC5 channels (Table 1). Moreover, AC1903 inhibited TRPV4 channels, but had limited effects on TRPV1 channels or Piezo1 channels. Discrepancies between some of our results and earlier reports on AC1903 could be related to differences between experimental protocols. For example, we measured the slow accumulation of [Ca^2+^]_i_, whereas Zhou *et al.* performed rapid measurements of currents, which include significant monovalent cation fluxes not detected in [Ca^2+^]_i_ recordings. In addition, Zhou *et al.* measured *acute* inhibition of conductivity of TRPC4, TRPC5, and TRPC6 channels by AC1903. Channels were activated with riluzole (TRPC5), carbachol (TRPC4), and OAG (TRPC6), respectively, and effects of AC1903 on activated channels were measured for ∼20 s before AC1903 was washed out again. In contrast, we preincubated cells expressing the relevant TRPC proteins with AC1903 for 30 min prior to addition of the relevant activator (EA or S1P for TRPC4:C4 and TRPC5:C5; EA for TRPC4–C1 and TRPC5–C1; OAG for TRPC3:C3 and TRPC6:C6; 4α-PDD for TRPV4:V4; Yoda1 for Piezo1). To address such differences, we performed whole-cell patch-clamp recordings, which showed acute, concentration-dependent, and reversible inhibition of EA-activated TRPC4:C4 channels by AC1903. Zhou *et al.* may have failed to observe clear inhibition of TRPC4 and TRPC6 channels by AC1903 because their compound application periods were short and their currents were relatively small and already decaying at the time of AC1903 application. We suggest that longer equilibration times may be needed to see inhibition of these channel types.

High-quality chemical probes are powerful tools in target validation studies and can serve as useful starting points for drug development, especially when combined with genetic approaches. In our recent reviews, we highlighted the use of small-molecule modulators of TRPC1/4/5 channels to complement genetic approaches in dissecting the different roles of specific TRPC1/4/5 channels across species, tissues, and pathologies (36, 37). However, even high-quality chemical probes are likely to have off-targets, and potency and selectivity of a chemical probe may be dependent on factors such as cellular context, mode of application, and treatment time. As part of the development of AC1903, the compound was screened against a standard kinase panel, in which no off-target effects were found (29). In cellular experiments, AC1903 is typically used as a TRPC5 inhibitor at concentrations of 30 to 100 μM (29, 56). However, our data suggest that TRPC3, TRPC4, TRPC6, and TRPV4 channels are important potential off-targets of AC1903 at these concentrations. In addition, the inhibition by AC1903 of the carbachol-mediated calcium response in wild-type HEK 293 cells (which lacked response to EA) suggests that AC1903 targets additional cellular calcium handling mechanisms. Therefore, data from experiments in which AC1903 is used as a TRPC5 inhibitor need to be interpreted with these results in mind, and additional chemical and genetic approaches may be required to dissect the roles of specific channels.

The effects of AC1903 on G418-mediate PTC readthrough could be reproduced with the broad-spectrum calcium channel inhibitors SKF96365 and 2-APB—which are known to target TRPC channels as well as other TRP channels—but not with selective inhibitors of TRPC1/4/5 (Pico145 or GFB-8438), TRPC3/6/7 (SAR7334), TRPC1/3/4/5/6/7 (GFB-8438 + SAR7334), or TRPV4 (GSK2193874). Therefore, it is possible that AC1903 suppresses PTC readthrough by inhibition of multiple cation channels involved in G418 uptake. This hypothesis is consistent with our pharmacological profiling of AC1903 and with previous reports suggesting that multiple nonselective cation channels (*e.g.*, TRPV1 and TRPV4) can mediate import of aminoglycosides (24, 25, 28). The potential for direct permeation of small organic cations through the pore of nonselective cation channels (*e.g.*, P2X (63, 64, 65, 66), TRPV1 (25, 28, 67, 68, 69, 70), TRPV2 (71, 72, 73), TRPV4 (24, 28), and TRPA1 (74)) is a topic of multiple investigations (75). In addition, a recent cryo-EM structure of a TRPV2 channel showed that doxorubicin (an organic cation at physiological pH) can enter the TRPV2 pore in the presence of the TRPV2 activator 2-APB (76). Determination of any specific TRP channels involved in the suppression of G418 uptake by AC1903 (as well as SKF96365 and 2-APB) is challenging. TRP proteins (especially those of the TRPC subfamily) can form various homo- and heterotetrameric channels with distinct biophysical properties, localization, pharmacology, and function, and genetic perturbation of TRP proteins (knockout or knockdown) can cause alterations of channel stoichiometries as well as compensatory upregulation of other (TRP) ion channels. We considered further investigation of the mode of action of AC103 by siRNA-based silencing of specific TRP proteins. However, the abundance of endogenous TRPCs (and other TRPs) is generally quite low and often at the limits of reliable detection by biochemical or functional assays. Therefore, ensuring efficient knockdown of specific TRP subunits is technically challenging, limiting the chance of obtaining meaningful results. In addition, it remains possible that AC1903 suppresses G418-induced PTC readthrough *via* other mechanisms than TRP channel inhibition.

The potential implication of the R175H mutation near the putative zinc binding site of TRPC5—which also incorporates cysteines shown to undergo S-glutathionylation and S-palmitoylation—in suppression of G418-induced PTC readthrough is currently not understood. In addition, it is difficult to explain why a small difference in intracellular G418 concentrations between DMS-114 and TC-71 cells would result in such profound differences in formation of PTC readthrough product, suggesting that additional cellular mechanisms may be involved. Further work is required to determine if, and how, the R175H mutation affects TRPC5 channel localization and function. This mutation of *TRPC5* is hemizygous in the TC-71 cell line, further increasing the number of potential homo- and heteromeric channels that could incorporate TRPC5.

In conclusion, our work reveals that AC1903 is not a selective TRPC5 inhibitor. However, the finding that AC1903 suppresses G418 uptake and G418-induced PTC readthrough—mimicking the broad-spectrum calcium channel modulators SKF-96365 and 2-APB—strengthens the hypothesis that various nonselective calcium channels are involved in aminoglycoside uptake. Further studies are required to understand the roles of specific TRP channels in aminoglycoside uptake in different cell types. Such understanding may guide studies of PTC readthrough in cells and animal models to determine the therapeutic potential of aminoglycosides in the treatment of rare genetic diseases. For example, the supplementation of aminoglycosides with specific TRP channel agonists may result in more selective targeting of cells, potentially leading to better therapeutic windows.

## Experimental procedures

### Human cells for PTC readthrough

DMS-114 and TC-71 cell lines with homozygous nonsense mutation in the *TP53* gene (NM 000546.5:c.637 C > T; NP 000537.3:p.R213X) were purchased from the American Type Culture Collection and the German Collection of Microorganisms and Cell Cultures, respectively. DMS-114 and TC-71 cells were cultured in RPMI-1640 medium (Sigma-Aldrich) supplemented with 10% (vol/vol) FBS and 1% antibiotic-antimycotic at 37 °C and 5% (vol/vol) CO_2_. Immortalized JEB01 keratinocytes were derived from a JEB patient with a homozygous nonsense mutation in the *COL17A1* gene (NM_000494.4:c.2062C > T; NP_000485.3:p.R688X) (77). JEB01 keratinocytes were cultured in defined keratinocyte serum-free medium (K-SFM) supplemented with defined K-SFM growth supplement (Gibco/Thermo Fisher Scientific) and 1% antibiotic–antimycotic (Gibco/Thermo Fisher Scientific, MD, USA) at 37 °C and 5% (vol/vol) CO_2_.

### Cell lines for TRP channel assays and viability studies

HEK T-REx cells expressing tetracycline-inducible TRPC4, TRPC5, TRPC5–C1,TRPC4–C1, and hPiezo1 have been described previously (46, 57, 59, 78). Cloning of TRPC3 has been described previously (79). To create cells stably expressing tetracycline-inducible TRPC3, HEK T-REx cells were transfected with pcDNA4/TO/hTRPC3 using jetPRIME transfection reagent (VWR) according to manufacturer’s instructions. After 48 h cells were put under antibiotic selection using 400 μg/ml zeocin and 10 μg/ml Blasticidin S (Invivogen)). Medium changes were carried out every 2 to 3 days to remove dead cells. Inducible HEK T-REx cells were cultured in Dulbecco’s modified Eagle medium GlutaMAX (Thermo Fisher Scientific) containing 10% fetal bovine serum (FBS; Merck), penicillin-streptomycin (100 units ml^−1^/100 μg ml^−1^), with the addition of blasticidin (10 μg ml^1^; Invivogen) and zeocin (Invivogen) at either 400 μg ml^−1^ (TRPC cell lines) or 200 μg ml^−1^ (hPiezo1) to maintain the stable incorporation of the tetracycline repressor and the channel of interest, respectively. Expression of tetracycline-inducible proteins was induced by the addition of tetracycline (TRPC cell lines: 1 μg ml^−1^, hPiezo1: 100 ng ml^−1^; 24 h) to cell culture medium. Experiments with TRPC6 and TRPV1 were carried out in WT HEK 293 cells transiently transfected with hTRPC6 (58) or rat TRPV1 (plasmid donated by Prof. Nikita Gamper, University of Leeds). WT HEK 293 cells were maintained in DMEM containing 10% FBS and penicillin-streptomycin (100 units ml^−1^/100 μg ml^−1^). Cells were transfected with hTRPC6 (cloned into pcDNA3) or rat TRPV1 using jetPRIME transfection reagent (VWR). Cells were assayed 48 h after transfection. CHO cells stably expressing TRPV4 (46) were cultured in Ham’s Nutrient Mixture F12 (Thermo Fisher Scientific), supplemented with 10% FBS, penicillin-streptomycin (100 units ml^−1^/100 μg ml^−1^), and 1 mg ml^−1^ G418 (Invivogen) to maintain expression of TRPV4. A498 cells were cultured in Minimum Essential Medium (MEM) with Earle’s salts (Thermo Fisher Scientific) supplemented with 10% fetal bovine serum and penicillin-streptomycin (100 units ml^−1^/100 μg ml^−1^).

### Automated capillary electrophoresis western analysis

The Western analysis assays for p53, Vinculin, and GAPDH detection were performed as previously described (18). Briefly, mixtures of 1 mg ml^−1^ cell lysates and the fluorescent master mix were heated at 95 °C for 5 min. The samples and all other reagents were dispensed into the microplates and capillary electrophoresis Western analysis was carried out with the ProteinSimple WES instrument and analyzed using the inbuilt Compass software (ProteinSimple). DO-1 mouse anti-p53 antibody (1:400, Santa Cruz sc-126), mouse anti-vinculin antibody (1:600, R&D Systems MAB6896), and rabbit anti-GAPDH (1:800, Abcam ab128915) were used.

### SDS-PAGE and immunoblotting

Human JEB01 keratinocytes were seeded into 6-well tissue culture dishes and exposed to indicated compounds. After 24 h, cells were lysed and 15 μg total protein from each lysate was separated on a 6% polyacrylamide gel, electrotransferred onto a nitrocellulose membrane, blocked, incubated with rabbit anti-Collagen XVII antibody (1:1,000, Abcam ab184996) overnight at 4 °C, washed and incubated with HRP-conjugated goat anti-rabbit secondary antibody, and developed using enhanced chemiluminescence substrate (Millipore). Membrane was stripped using 0.1 N NaOH and reprobed with rabbit anti-beta actin antibody (1:10,000, Novus Biologicals) and detected as above.

### Measurement of intracellular G418

Intracellular G418 was measured in cell lysates prepared for Western analysis using an indirect competitive gentamicin ELISA kit (Creative Diagnostics, DEIA047), according to the manufacturer’s protocol, as previously described (80).

### Intracellular Ca^2+^ measurements

[Ca^2+^]_i_ recordings were carried out using the ratiometric Ca^2+^ dye Fura-2. 24 h prior to experiments, cells were plated either onto black, clear bottom poly-d-lysine-coated 96-well plates (HEK cells) or clear 96-well plates (A498 cells, CHO cells) at 50,000 cells per well (HEK cells) or 20,000 cells per well (A498 cells, CHO cells). For tetracycline-inducible HEK T-REx cells, protein expression was induced with tetracycline (TRPC channels: 1 μg ml^−1^; hPiezo1: 100 ng ml^−1^) at this point. To load cells with the Fura-2 dye, media was removed and cells were incubated with standard bath solution (SBS) containing 2 μM Fura-2 acetoxymethyl ester (Fura-2 AM; Thermo Fisher Scientific) and 0.01% pluronic acid for 1 h at 37 °C. SBS contained (in mM): NaCl 130, KCl 5, glucose 8, HEPES 10, MgCl_2_ 1.2 and CaCl_2_ 1.5. After this incubation, cells were washed twice with fresh SBS. SBS was then changed to recording buffer, which consisted of SBS with 0.01% pluronic acid and the relevant concentration of AC1903, diluted in DMSO (final DMSO concentration: 0.01%). Control wells contained SBS with 0.01% pluronic acid and 0.01% DMSO. Cells were then incubated for 30 min prior to the experiment. For experiments with CHO cells, probenecid at 2.5 mM was included throughout the experiment to prevent extrusion of Fura-2 from the cells. Measurements were carried out using a FlexStation (Molecular Devices), using excitation wavelengths of 340 and 380 nm, at an emission wavelength of 510 nm. [Ca^2+^]_i_ recordings were performed at room temperature at 5 s intervals for 300 s. Compounds were added from a compound plate at 2x the final concentration after recording for 60 s.

### Whole-cell patch-clamp recordings

For electrophysiology experiments, cells were plated at a low density of 20 to 30% onto round coverslips (13 mm diameter or 5 × 5 mm), and TRPC4 expression in HEK T-REx was induced with 1 μg ml^−1^ tetracycline 24 h before experimentation. Experiments were carried out at room temperature. Patch-clamp recordings were performed in whole-cell mode under voltage clamp at room temperature using 3 to 5 MΩ patch pipettes fabricated from borosilicate glass capillaries with an outside diameter of 1 mm and an inside diameter of 0.58 mm (Harvard Apparatus). The voltage protocol comprised voltage ramps applied from -100 to +100 mV every 10 s from a holding potential of 0 mV. The patch-clamp currents were recorded using an Axopatch 200B amplifier, digitized by a Digidata 1440 and recorded to a computer using pCLAMP10 (Molecular Devices). The data were filtered at 1 kHz and analyzed offline using Clampfit 10.7 software and Origin 2019b software (OriginLab). The bath solution consisted of SBS and the pipette solution (intracellular solution) contained (in mM) CsCl 145, MgCl_2_ 2, HEPES 10, EGTA (free acid) 1, ATP (sodium salt) 5, NaGTP 0.1, titrated to pH 7.2 with CsOH. All solutions were filtered using a 0.2 μm filter (Nalgene Rapid Flow, Thermo Scientific). Upon whole cell configuration, cells were continually perfused at a rate of 2 ml ml^−1^ with SBS containing 0.01% pluronic acid, followed by SBS +0.01% pluronic acid + EA (30 nM).

### Cell viability assay

Cell viability assays were carried out using WST-1 Cell Proliferation Reagent (Merck). A498 cells were plated onto clear 96-well plates at 4000 cells per well and left to adhere for 24 h. Media was then removed, and replaced with fresh media containing 100 nM EA, in presence of either DMSO or 0.1 to 100 μM AC1903. Control wells were treated with vehicle (DMSO) only. To exclude any potential cytotoxic effects of AC1903 itself, cells were incubated with 0.1 to 100 μM AC1903 in absence of EA. Cells were treated in triplicate. Cells were incubated with compounds for 8 h. After 8 h, treatment media was removed and media containing WST-1 reagent (10 μl reagent +100 μl media per well) was added to each well and incubated for 1.5 h at 37 °C. Blank wells contained media and WST-1 reagent (no cells). After incubation, the plate was shaken for 1 min. Absorbance was then read at 440 nm and 650 nm (reference wavelength). To analyze data, the value of the 650 nm was subtracted from that of the 440 nm reading. Values across triplicate wells were averaged, and the value from the blank wells subtracted to account for background absorbance. Values from compound-treated cells were then compared with control cells (DMSO-treated).

### Data analysis

For G418 uptake experiments, statistical analysis was performed using GraphPad Prism 8.0. One-way or two-way Analysis of Variance (ANOVA) was used to analyze the difference between different treatments or samples as stated in figure legends. Differences were considered significant at a *p* value of <0.05.

For Ca^2+^ recording, electrophysiology, and cell viability experiments, data were analyzed using GraphPad Prism 9.0. Representative data are presented as raw data. Unless indicated otherwise, experiments were carried out at n = 3, where n = number of independent experiments. Each independent FlexStation experiment consisted of six technical replicates (*i.e.*, six wells of a 96-well plate for each Flex Station assay), unless stated otherwise. Concentration–response curves were fitted in GraphPad Prism using a four-parameter curve fit. Amplitudes of Ca^2+^ responses for different channels were measured at time points indicated in the corresponding figure legends. For cell viability data, one-way ANOVA with Šídák multiple comparisons test was used to analyze the difference between treatments. For electrophysiology data, paired *t* test was used to analyze the difference between responses in absence and presence of AC1903. Differences were considered significant at a *p* value of <0.05.

### Absorbance and fluorescent properties of AC1903

AC1903 or vehicle (DMSO) was dissolved in SBS to the stated concentration and added to wells of a clear 96-well plate. Spectra were recorded using a FlexStation. Absorbance was recorded from 200 to 850 nm at 10 nm intervals. A fluorescence excitation spectrum was recorded from 250 to 800 nm at 10 nm intervals with emission fixed at 510 nm (the emission wavelength of Fura-2). Fluorescence emission spectra were recorded from 250 to 800 nm at 10 nm intervals, with excitation fixed at either 340 nm or 380 nm (excitation wavelengths for Fura-2).

### Chemicals

(-)-Englerin A (EA) was obtained from PhytoLab, AC1903 was obtained from Cayman Chemical, and identity and purity were confirmed through analysis by ^1^H NMR, ^13^C NMR, high-resolution mass spectrometry, and HPLC analysis (Figs. S1–S4); data were consistent with previous reports (30). 1-oleoyl-2-acetyl-sn-glycerol (OAG) and 4α-phorbol 12,13-didecanoate (4α-PDD) were obtained from Merck. Sphingosine-1-phosphate (S1P) and capsaicin were obtained from Bio-techne. Carbachol was obtained from Alfa Aesar Chemicals. Yoda1 and SAR7334 were obtained from Tocris Bioscience. GSK2193874 was obtained from Sigma-Aldrich. SKF96365 and 2-APB were obtained from Insight Biotechnology Ltd. Pico145 (46) and GFB-8438 (44) were synthesized according to literature procedures and purified to homogeneity. Chemicals were made up as 10 mM (EA, 4α-PDD, Yoda-1, GFB-8438, SAR7334), 50 mM (GSK2193874, SKF96365), 100 mM (AC1903, OAG), or 300 mM (2-APB) stocks in 100% DMSO, aliquots of which were stored at −20 °C (Yoda1, AC1903, GFB-8438, SAR7334, GSK2193874, SKF96365, 2-APB) or −80 °C (EA, 4α-PDD, OAG)). S1P was made up in methanol to 5 mM and stored as aliquots at −80 ⁰C. Further dilutions of EA and AC1903 were made in DMSO if required, and these were dissolved 1:1000 in recording and compound buffer (SBS +0.01% pluronic acid) before being added to cells. Fura-2-AM (Thermo Fisher Scientific) was dissolved at 1 mM in DMSO.

## Data availability

Data relating to this manuscript are available from the corresponding authors upon reasonable request.

## Supporting information

This article contains supporting information.

## Conflict of interest

David J. Beech is an inventor on the following patent applications: (1) PCT/GB2018/050369. TRPC ion channel inhibitors for use in therapy. Filing date: 9th February 2018. Inventors: David J. Beech, Richard J. Foster, Sin Ying Cheung and Baptiste M Rode; (2) 62/529,063. Englerin derivatives for the treatment of cancer. Filing date: 6th July 2017. Inventors: John A. Beutler, Antonio Echavarren, William Chain, David Beech, Zhenhua Wu, Jean-Simon Suppo, Fernando Bravo, and Hussein Rubaiy.
