# Supplementary material for: Nonselective TRPC channel inhibition and suppression of aminoglycoside-induced premature termination codon readthrough by the small molecule AC1903
Source: J Biol Chem. 2022 Jan 6;298(2):101546. doi: 10.1016/j.jbc.2021.101546 (PMC8808171; doi:10.1016/j.jbc.2021.101546)
Supplement: Supplemental Figures S1–S12 [file mmc1.pdf]

Supporting Figures for:

Non-selective TRPC channel inhibition and suppression of aminoglycoside-induced premature termination codon readthrough by the small molecule AC1903

Alireza Baradaran-Heravi,[1,†,*] Claudia C. Bauer,[2,†] Isabelle B. Pickles,[2,3] Sara Hosseini-Farahabadi,[1] Aruna D. Balgi,[1] Kunho Choi,[1] Deborah M. Linley,[2] David J. Beech,[2] Michel Roberge,[1] Robin S. Bon[2,4,*]

[1] Department of Biochemistry and Molecular Biology, Life Sciences Institute, The University of British Columbia, Vancouver, British Columbia, Canada V6T 1Z3.

[2] Discovery and Translational Science Department, Leeds Institute of Cardiovascular and Metabolic Medicine, School of Medicine, University of Leeds, Leeds LS2 9JT, UK.

[3] School of Chemistry, University of Leeds, Leeds LS2 9JT, UK.

[4] Astbury Centre for Structural Molecular Biology, University of Leeds, Leeds LS2 9JT, UK.

* Correspondence should be addressed to R.S.B. (r.bon@leeds.ac.uk) or A.B.-H. (abara@mail.ubc.ca)

† These authors contributed equally.

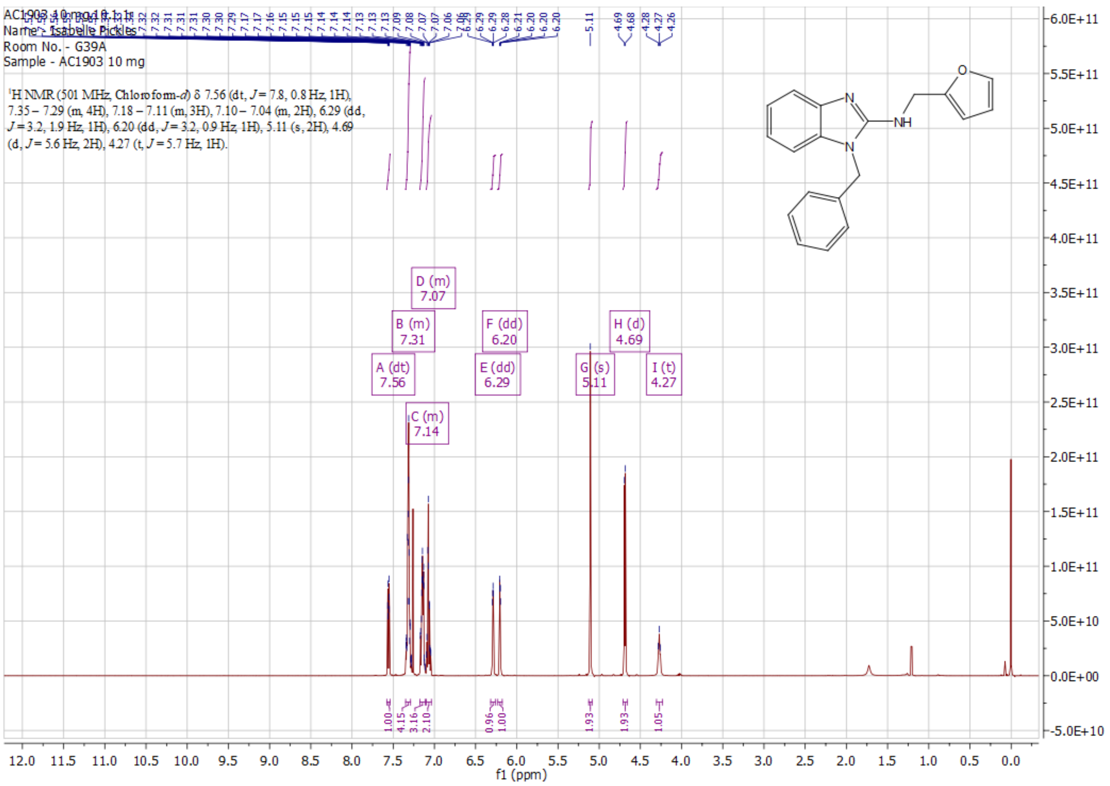

Figure S1. ^{1}H NMR spectrum of commercial AC1903 (Cayman Chemical, Ann Arbor, USA).

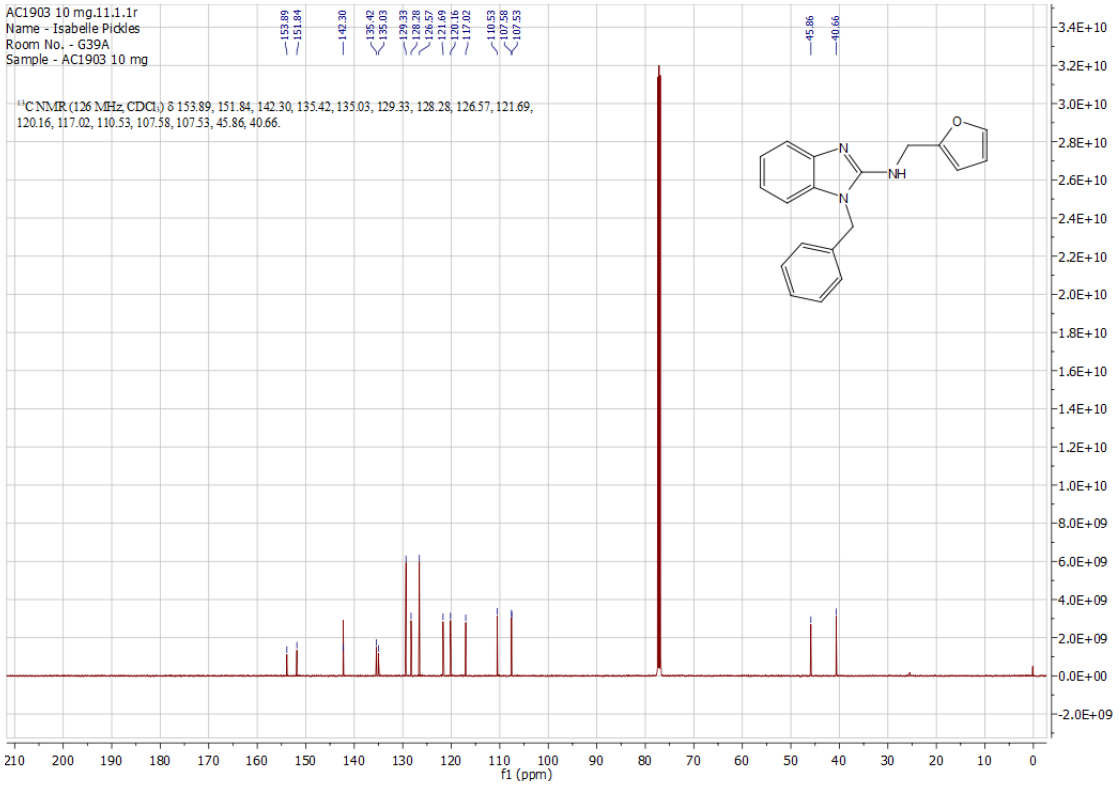

Figure S2. ^{13}C NMR spectrum of commercial AC1903 (Cayman Chemical, Ann Arbor, USA).

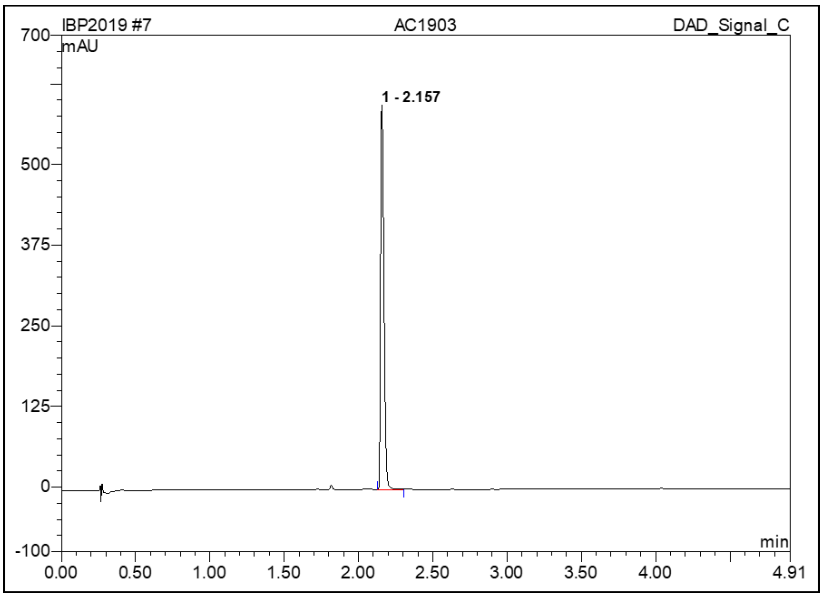

No.	Ret.Time min	Peak Name	Height mAU	Area mAU*min	Rel.Area %	Amount	Type
1	2.16	n.a.	595.121	15.256	100.00	n.a.	BMB
Total:			595.121	15.256	100.00	0.000	

Figure S3. HPLC trace of commercial AC1903 (Cayman Chemical, Ann Arbor, USA). Analysis was performed on an Agilent 1290 Infinity Series equipped with a UV detector (set at 254 nm) and a Hyperclone C18 reverse phase column using MeCN/water (5→95% or 50→95%) containing 0.1% formic acid, at 0.5 mL min^{-1} over a period of five minutes.

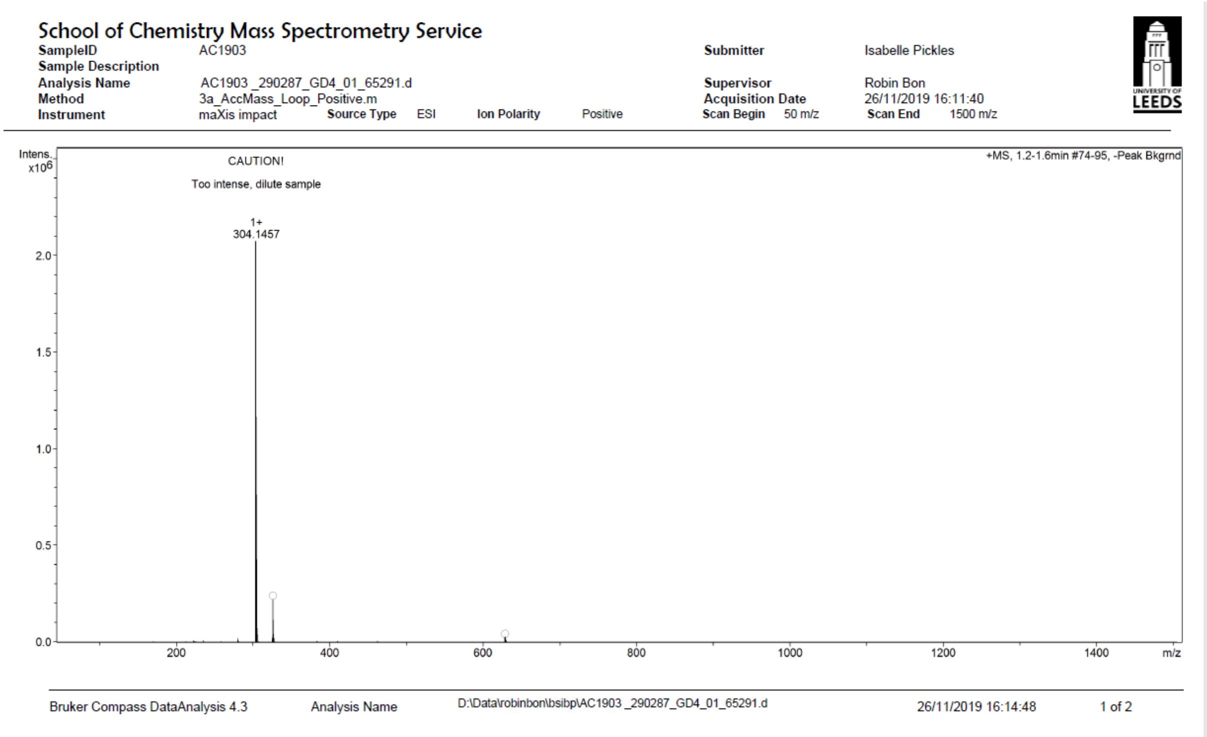

Figure S4. HRMS spectrum of commercial AC1903 (Cayman Chemical, Ann Arbor, USA).

1-Benzyl-N-(furan-2-ylmethyl)-1H-benzo[d]imidazol-2-amine, AC1903 (Cayman Chemical, Ann Arbor, USA) ^{1}H NMR (501 MHz, Chloroform-d) δ 7.56 (dt, J = 7.8, 0.8 Hz, 1H), 7.35 – 7.29 (m, 4H), 7.18 – 7.11 (m, 3H), 7.10 – 7.04 (m, 2H), 6.29 (dd, J = 3.2, 1.9 Hz, 1H), 6.20 (dd, J = 3.2, 0.9 Hz, 1H), 5.11 (s, 2H), 4.69 (d, J = 5.6 Hz, 2H), 4.27 (t, J = 5.7 Hz, 1H); ^{13}C NMR (126 MHz, CDCl3) δ 153.9, 151.8, 142.3, 135.4, 135.0, 129.3, 128.3, 126.6, 121.7, 120.2, 117.0, 110.5, 107.6, 107.5, 45.9, 40.7; ESI-HRMS: calc. for $C_{19}H_{17}N_3NaO$ $[M+Na]^+$ 326.1264, found 326.1263; ESI-LCMS: m/z 304.21 $[M+H]^+$; HPLC (5-95 % MeCN in water) RT = 2.16 min.

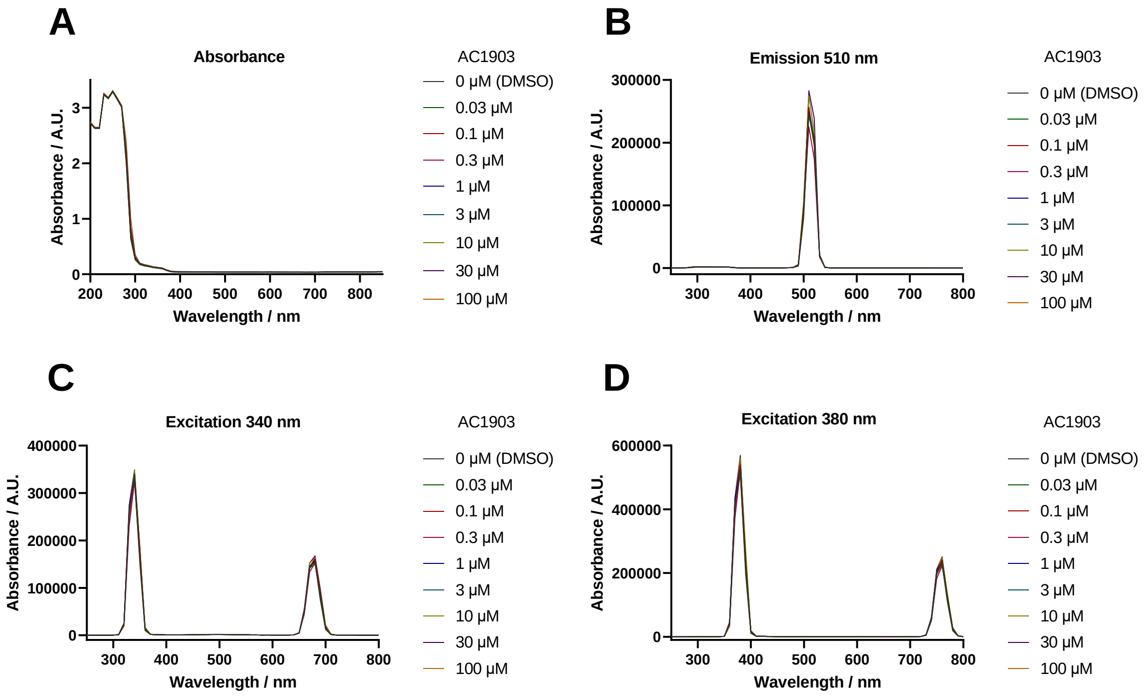

Figure S5. Absorbance and fluorescence spectra of solutions containing AC1903 (Cayman Chemical, Ann Arbor, USA). A) Absorbance spectra of SBS containing DMSO or 0.03-100 µM AC1903. Absorbance was measured at 200-850 nm at 10 nm intervals. B) Excitation spectra of SBS containing DMSO or 0.03-100 µM AC1903. Emission was fixed at 510 nm, with excitation of 250-800 nm at 10 nm intervals. C,D) Fluorescence emission spectra of SBS containing DMSO or 0.03-100 µM. Excitation wavelength was fixed at either 340 nm (C) or 380 nm (D), while emission was measured at 250-800 nm, at 10 nm intervals.

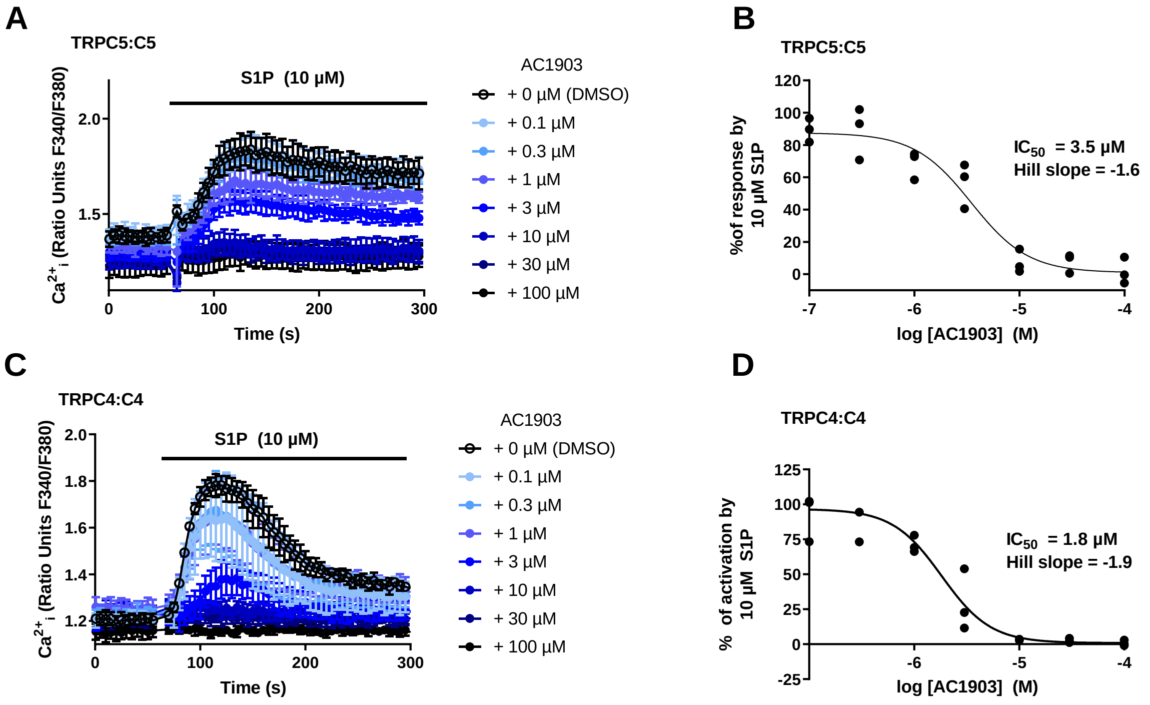

Figure S6. AC1903 inhibits S1P-mediated activation of TRPC5:C5 and TRPC4:C4 channels. A,C) Representative $[Ca^{2+}]_i$ measurements from a single 96-well plate (N = 6; mean ± SD over technical replicates) showing inhibition of 10 µM S1P-mediated $[Ca^{2+}]_i$ responses by 0.1-100 µM AC1903 in (Tet+) HEK T-REx cells expressing TRPC5 (A) or TRPC4 (C). B,D) Concentration-response data for experiments in (A) and (C), respectively (scatter plots showing normalised data for three independent experiments; n/N = 3/18). Responses were calculated at 100-150 s compared to $[Ca^{2+}]_i$ at baseline (0-55 s).

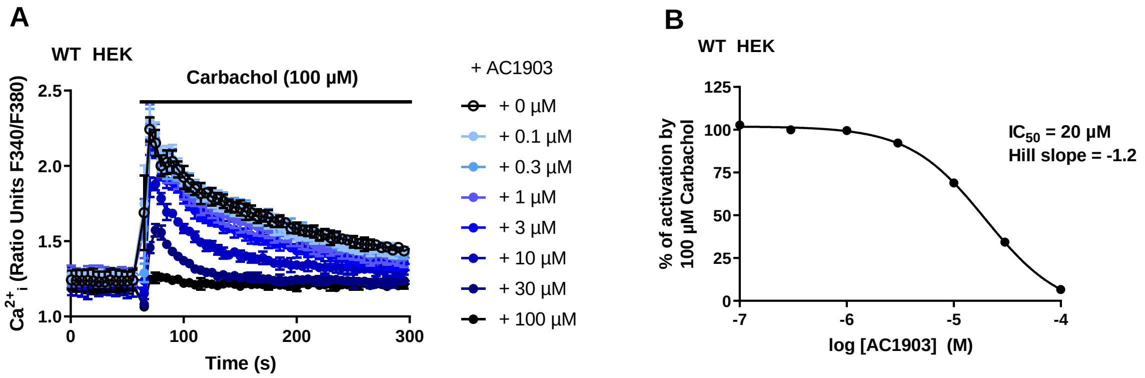

Figure S7. AC1903 inhibits carbachol-mediated [Ca^{2+}]$_i$ responses in WT HEK 293 cells. A) [Ca^{2+}]$_i$ measurements from a single 96-well plate (N = 6; mean ± SD over technical replicates) showing inhibition of carbachol-mediated [Ca^{2+}]$_i$ responses by 0.1-100 µM AC1903 in WT HEK 293 cells. B) Concentration-response data for experiment in (A), showing mean responses from six technical repeats (n/N = 1/6). Responses were calculated at 70-80 s compared to [Ca^{2+}]$_i$ at baseline (0-55 s).

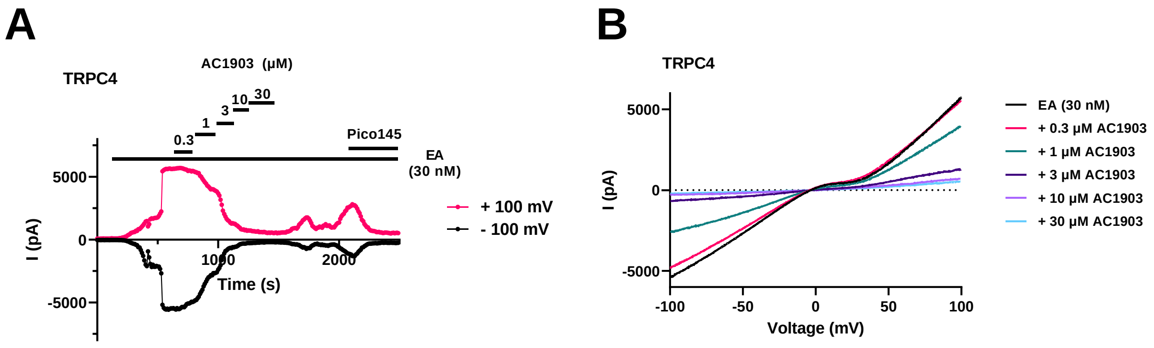

Figure S8. Inhibition of EA-activated TRPC4 currents in whole-cell patch-clamp recordings by AC1903 is concentration-dependent. A) Trace from one (Tet+) HEK T-REx cell expressing TRPC4, showing current at +100 mV (magenta) and -100 mV (black) after activation by EA (30 nM), followed by cumulative addition of AC1903 (0.3-30 µM). B) Current-voltage relationships for experiment in (A).

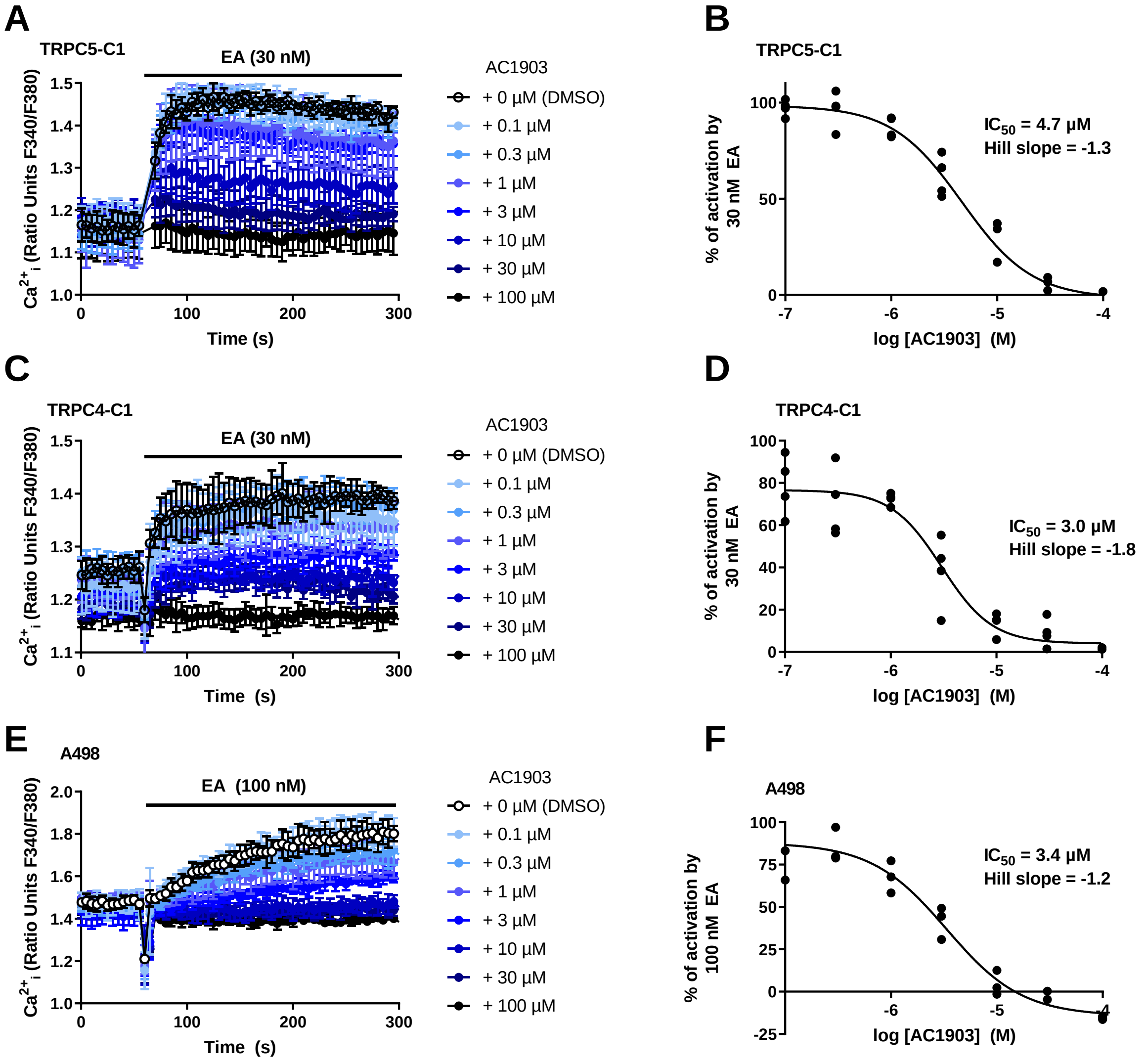

Figure S9. AC1903 inhibits heteromeric TRPC1/4/5 channels. A, C) Representative $[Ca^{2+}]_i$ measurements from a single 96-well plate (N = 6; mean ± SD over technical replicates) showing inhibition of EA-mediated $[Ca^{2+}]_i$ responses by 0.1-100 µM AC1903 in (Tet+) HEK T-REx cells expressing concatemeric TRPC5-C1 (A) or concatemeric TRPC4-C1 (C). B,D) Concentration-response data for experiments in (A) and (C) (scatter plots showing normalised data for three independent experiments; n/N = 3-4/18-24). Responses were calculated at 250-295 s compared to $[Ca^{2+}]_i$ at baseline (0-55 s). E) Representative Ca^{2+} measurements from a single 96-well plate (N = 6; mean ± SD over technical replicates) showing inhibition of EA-mediated $[Ca^{2+}]_i$ responses by 0.1-100 µM AC1903 in A498 cells. F) Concentration-response data for experiments in (E) (scatter plot showing normalised data for three independent experiments; n/N = 3/17-18). Responses were calculated at 250-300 s compared to $[Ca^{2+}]_i$ at baseline (0-55 s).

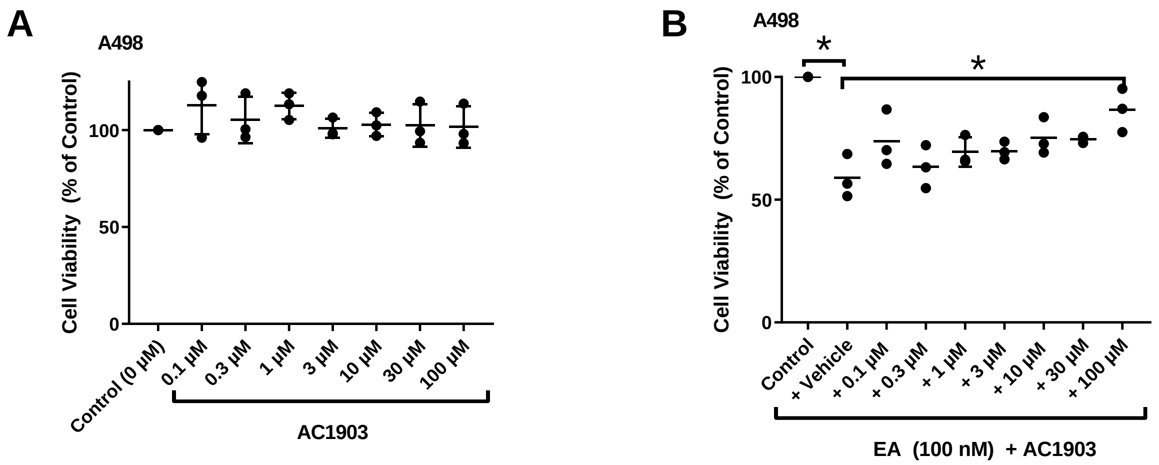

Figure S10. AC1903 is not toxic to A498 renal cancer cells but (at 100 µM) inhibits EA-mediated A498 cytotoxicity. A) Cell viability data for A498 cells treated with DMSO (control) or 0.1-100 µM AC1903 for 8 hours. Cell viability was calculated as % of control, and the effect of AC1903 was compared to control (0 µM) (scatter plots showing data from three independent experiments; bars represent mean ± SD; n = 3). B) Cell viability data for A498 cells treated with DMSO (Control) or 100 nM EA in combination with either vehicle (DMSO) or 0.1-100 µM AC1903 for 8 hours. Cell viability was calculated as % of control, and the effect of AC1903 was compared to EA + vehicle (scatter plots showing data from three independent experiments; bars represent mean ± SD; n = 3). * indicates statistically significant difference between samples according to one-way ANOVA with Šídák multiple comparisons test ($P < 0.05$).

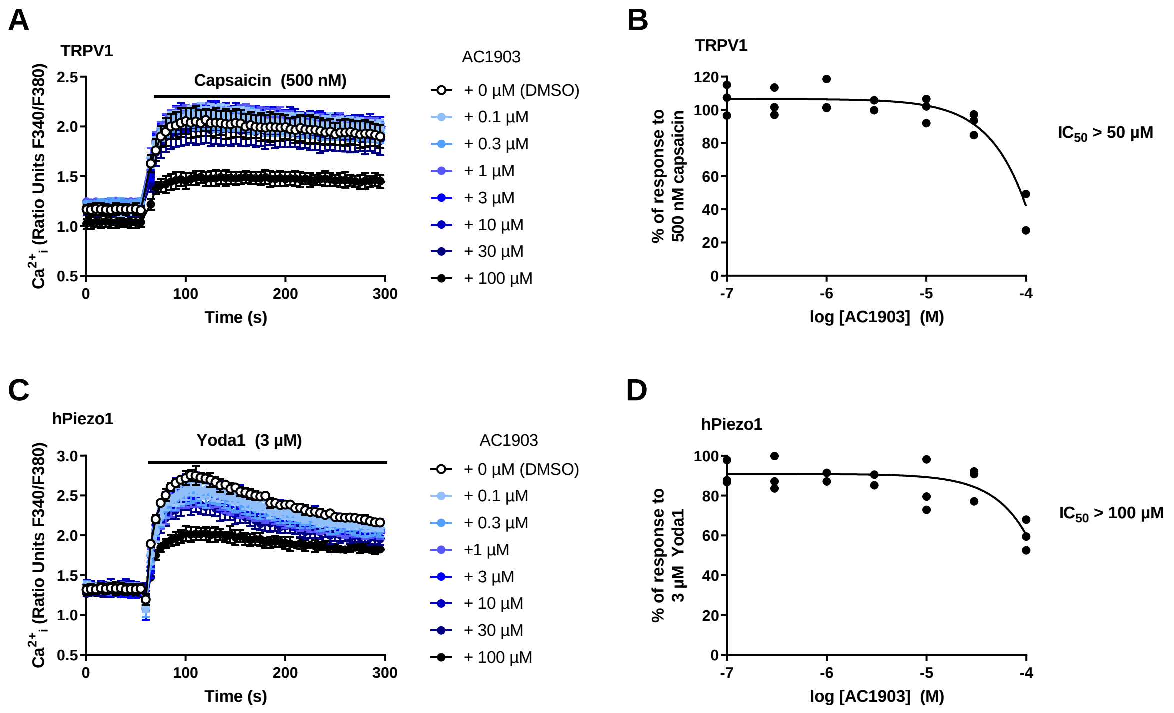

Figure S11. AC1903 (up to 30 µM) does not affect TRPV1 or hPiezo1 activation. A) Representative $[Ca^{2+}]_i$ responses from one 96-well plate (N = 6; mean ± SD over technical replicates) showing the effect of DMSO or 0.1-100 µM AC1903 on capsaicin-mediated activation of TRPV1 expressed in HEK 293 cells. B) Concentration-response data for experiments in (A) (scatter plot showing normalised data for three independent experiments; n/N = 3/18). Responses were calculated at 95-100 s, compared to baseline at 0-55 s. C) Representative $[Ca^{2+}]_i$ responses from one 96-well plate (N = 6; mean ± SD over technical replicates) showing the effect of DMSO or 0.1 -100 µM AC1903 on Yoda1-mediated activation in HEK T-REx (Tet+) cells expressing hPiezo1. D) Concentration-response data for experiments in (C) (scatter plot showing normalised data for three independent experiments; n/N = 3/18). Responses were calculated at 95-115 s compared to $[Ca^{2+}]_i$ at baseline (0-55 s).

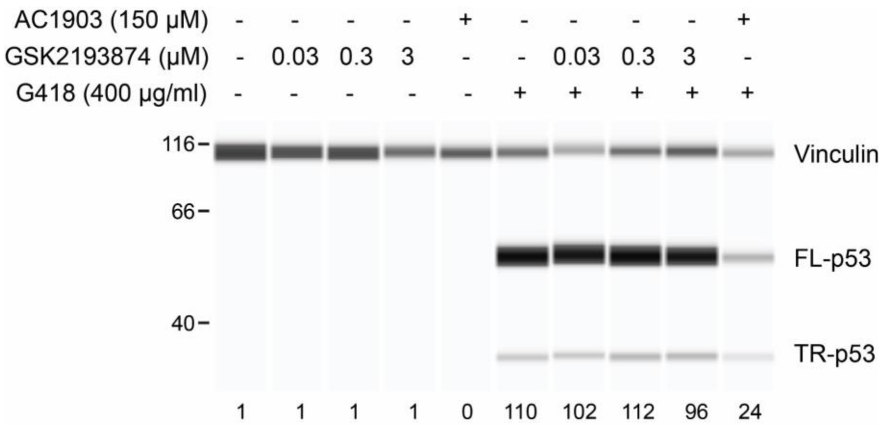

Figure S12. Effect of the TRPV4 inhibitor GSK2193874 on G418-induced PTC readthrough. DMS-114 cells were preincubated with AC1903 or indicated concentrations of the selective TRPV4 channel inhibitor GSK2193874 for 3 h followed by exposure to 400 µg/ml G418 for another 3 h. At 24 h cell lysates were prepared and p53 levels were measured by automated capillary electrophoresis western analysis (using vinculin as loading control). Samples of cell lysates containing equal amounts of total protein were loaded in all capillaries and FL-p53 levels were expressed relative to the amount of FL-p53 in untreated cells.
